# Multitask learning for early treatment response and survival prediction in lung cancer radiotherapy using sequential CBCT imaging

**DOI:** 10.3389/fonc.2026.1833763

**Published:** 2026-07-21

**Authors:** Yumei Li, Zhouji Wei, Chenlong Luo, Yejun Gong, Ye Zhang, Feng Zhang, Chunyan Liu

**Affiliations:** 1 The Second Affiliated Hospital of Guangxi University of Traditional Chinese Medicine, Nanning, China; 2 Department of Radiotherapy, Guangxi Zhuang Autonomous Region Ethnic Hospital, Nanning, China; 3 Affiliated Hengyang Hospital of Hunan Normal University & Hengyang Central Hospital, Hengyang, Hunan, China; 4 Key Laboratory of Computing and Stochastic Mathematics, School of Mathematics and Statistics, Hunan Normal University, Changsha, Hunan, China

**Keywords:** adaptive radiotherapy, cone-beam CT, multi-task learning, progression-free survival, radiomics, treatment response

## Abstract

**Background:**

Adaptive radiotherapy requires early prediction of treatment response and survival, yet the optimal use of longitudinal cone-beam computed tomography (CBCT) imaging and modeling strategy for small cohorts remains unclear.

**Purpose:**

To develop a multitask deep learning framework for simultaneous treatment response classification and progression-free survival (PFS) prediction using planning computed tomography (CT), dose, and sequential CBCT, and to evaluate the impact of including different numbers of early CBCT time points on prediction performance within an incremental analysis framework.

**Methods:**

A total of 142 lung cancer patients were retrospectively analyzed. A lightweight network with cross-modal attention fusion and dual task heads for treatment response classification and Cox-based survival prediction was trained end-to-end and benchmarked against 12 baseline methods via 5-fold cross-validation.

**Results:**

Using only the first on-treatment CBCT, the model achieved an area under the receiver operating characteristic curve (AUC) of 0.858 ± 0.078 and a concordance index (C-index) of 0.672 ± 0.058, outperforming all baselines. Ablation analysis confirmed the contributions of attention fusion and multitask training. Additional CBCT scans degraded classification performance, likely due to increased dimensionality relative to the cohort size.

**Conclusions:**

The first on-treatment CBCT provided the strongest early predictive signal for treatment response classification in this cohort; adding subsequent scans degraded classification performance and produced only a marginal change in survival concordance under the current study setting.

## 1 Introduction

Lung cancer remains the most frequently diagnosed cancer and the leading cause of cancer-related mortality worldwide, with 2.48 million new cases and 1.8 million deaths in 2022 (1). Definitive chemoradiotherapy is a cornerstone treatment for locally advanced non-small cell lung cancer (NSCLC), yet treatment response varies substantially: approximately 30%–50% of patients experience disease progression within 2 years despite aggressive multimodal therapy (2, 3). Early identification of patients at high risk of treatment failure would enable timely adaptation, including dose escalation, field modification, or addition of systemic agents. However, traditional prognostic models based on clinical variables and TNM staging fail to capture the biological heterogeneity that underlies individual treatment response (4).

Radiomics—the high-throughput extraction of quantitative imaging features—has emerged as a promising approach for non-invasive tumor phenotyping and personalized outcome prediction (5, 6). In lung cancer, CT-based radiomic signatures have demonstrated prognostic value for overall survival and recurrence following radiotherapy (7, 8). Beyond pretreatment imaging, delta-radiomics—tracking feature changes across serial scans—provides additional information about treatment-induced tumor dynamics. Fave et al. (9) showed that intratreatment CT radiomic features changed significantly during radiation therapy and that end-of-treatment texture features stratified recurrence risk in stage III NSCLC. Recent studies have further explored the predictive value of delta-radiomics in advanced NSCLC patients undergoing immunotherapy (10, 11). Cone-beam computed tomography (CBCT), routinely acquired during image-guided radiotherapy for positioning verification, offers an accessible source of longitudinal imaging without additional radiation burden (12). van Timmeren et al. (13) demonstrated that CBCT-derived radiomic signatures maintained prognostic value comparable to planning CT (C-index 0.66 vs. 0.69), establishing CBCT radiomics as a feasible monitoring tool. However, the number of early CBCT time points to include for prediction modeling and how best to integrate sequential imaging information remain open questions, especially in the context of treatment outcome prediction during radiotherapy (14, 15).

The rationale for extracting predictive features from the earliest on-treatment CBCT rests on the biology of early radiation response. Within the first 1 to 2 weeks of fractionated radiotherapy, tumors begin to exhibit measurable changes in density and volume that reflect the underlying radiosensitivity of the irradiated tissue, often before anatomic shrinkage becomes evident on conventional response assessment. Wen et al. (16) showed that CBCT-derived tumor density (CT number) and volume variations during chemoradiotherapy differed significantly between responders and non-responders in advanced NSCLC, with discriminative changes emerging in the early-to-mid treatment course. Mechanistically, early reductions in tumor CT number have been attributed to radiation-induced apoptotic cell loss and changes in tumor blood volume and microenvironment, and the magnitude of these imaging changes has been linked to intrinsic radiosensitivity; serial CT texture features likewise change measurably and dose-dependently during thoracic irradiation (16, 17). Consistent with this biology, early tumor shrinkage during concurrent chemoradiotherapy is an independent prognostic factor for both progression-free and overall survival in stage III NSCLC (18), and CBCT-based tumor regression during definitive chemoradiation is significantly associated with survival (19). Because standard RECIST assessment is typically performed 2 to 3 months after treatment, it cannot capture these early dynamics (16). The first on-treatment CBCT is particularly attractive in this regard: acquired in the actual treatment position, it captures the initial tumor burden together with the earliest treatment-related changes, providing a biologically grounded and clinically practical basis for early outcome prediction.

Recent advances have introduced increasingly sophisticated modeling approaches for treatment outcome prediction. DeepSurv (20) extended the Cox proportional hazards model with deep neural networks, achieving a C-index of 0.68 on large-scale datasets. CerviPro (21) fused pre- and post-treatment CT, radiomic features, and clinical variables through multimodal deep learning, reporting a C-index of 0.81 for cervical cancer prognostication. For longitudinal imaging, Jalalifar et al. (22) applied convolutional gated recurrent units (Conv-GRU) to sequential MRI for brain metastasis response prediction, demonstrating improved accuracy with additional follow-up scans. In lung cancer, specifically, Hosny et al. (7) trained a 3D CNN across seven independent datasets, achieving an AUC of 0.70 for a 2-year survival prediction. A common limitation of these methods is that they address classification or survival prediction in isolation. Multitask learning, which jointly models related outcomes through shared representations, has shown promise for improving performance in cancer prognosis (23, 24). Recent frameworks have integrated classification and survival tasks to enhance predictive stability in various cancers (25–27). Yet multitask approaches have rarely been applied to CBCT-based radiotherapy outcome prediction, and the integration of sequential CBCT features within a multitask deep learning framework remains unexplored. From a practical perspective, lightweight models are especially well suited to radiotherapy research, where longitudinal CBCT analysis can rapidly inflate feature dimensionality while cohort sizes often remain limited. In this setting, parsimonious models may offer greater robustness, facilitate clinical deployment, and improve reproducibility.

This study addresses these gaps by proposing a lightweight multitask deep learning framework for the joint prediction of treatment response and progression-free survival using planning CT, dose, and CBCT with cross-modal attention, by investigating how the number of early CBCT time points used as model inputs affects prediction performance through incremental analysis, and by benchmarking the proposed method against state-of-the-art multimodal deep learning and survival analysis models. The proposed framework consists of a shared encoder and two task-specific heads for classification and Cox-based survival prediction, trained end-to-end with joint optimization. We hypothesized that the earliest on-treatment CBCT would carry the dominant early predictive signal and that, given the moderate cohort size, appending additional sequential scans would increase input dimensionality faster than it adds independent information. This hypothesis, together with the competitiveness of the proposed framework relative to the baselines, is examined quantitatively in Section 3.

## 2 Materials and methods

### 2.1 Patient population and data collection

This retrospective study was conducted at Affiliated Hengyang Hospital and included 142 patients with histologically confirmed lung cancer who received definitive thoracic radiotherapy between January 2019 and December 2023. The study was approved by the institutional review board, and the requirement for informed consent was waived due to the study’s retrospective nature. Inclusion criteria were pathologically confirmed lung cancer, planned definitive radiotherapy with or without systemic therapy, availability of planning CT, radiation treatment plan, and at least one CBCT scan during treatment, as well as complete clinical and follow-up data. Patients with prior thoracic radiotherapy, incomplete imaging data, loss to follow-up within 6 months of treatment completion, or concurrent malignancies were excluded. During the study period, consecutive patients treated with definitive thoracic radiotherapy were screened; those who satisfied all inclusion criteria and none of the exclusion criteria, and for whom complete planning CT, treatment plan/dose, at least one on-treatment CBCT, and follow-up data were available, constituted the final analytic cohort of 142 patients. All patients had locally advanced (stage III) disease and were treated with conventionally fractionated thoracic radiotherapy (planned prescription dose 50–66 Gy, most commonly 60 Gy in 30 fractions; further detailed in Section 2.2). Follow-up was performed according to institutional practice with periodic clinical and imaging assessment after treatment completion.

Clinical variables collected included age, sex, Eastern Cooperative Oncology Group (ECOG) performance status, tumor histology, TNM stage (8th edition), T stage, N stage, systemic treatment regimen, radiation dose and fractionation, number of available CBCT scans, treatment response, and PFS. Tumor location and laterality were extracted from diagnostic and pathological records where available. Pretreatment evaluation generally included pathological confirmation, diagnostic chest CT or contrast-enhanced CT, routine staging workup, and clinical assessment before radiotherapy. Pretreatment positron emission tomography/computed tomography (PET/CT), smoking history, driver mutations, genetic alterations, and protein-expression biomarkers such as programmed death-ligand 1 (PD-L1) expression were not systematically available or consistently recorded for all patients in this retrospective cohort. Therefore, these variables were not included in model development or statistical analysis.

Treatment response was assessed using Response Evaluation Criteria in Solid Tumors version 1.1 (RECIST 1.1) at approximately 3 months after treatment, classifying patients as responders (complete or partial response) or non-responders (stable or progressive disease). PFS was defined as the time from treatment initiation to disease progression or death from any cause, with censoring at last follow-up; the median follow-up duration was 47.3 months (range: 6–84 months). Patient demographics and clinical characteristics are summarized in **Table 1**; the descriptive comparison of these characteristics between responders and non-responders is reported in the Results (Section 3.1).

**Table 1:** Summary of clinical data for the total cohort.

Characteristic	Total cohort (n = 142)	Responders (n = 102)	Non-responders (n = 40)	P-value
**Age, years**				0.166a
Mean ± SD	63.5 ± 7.5	63.1 ± 8.1	64.8 ± 5.8	
Median (range)	64 (42–79)	64 (42–79)	63 (49–76)	
**Sex, n (%)**				1.000b
Male	134 (94.4)	96 (94.1)	38 (95.0)	
Female	8 (5.6)	6 (5.9)	2 (5.0)	
**ECOG PS, n (%)**				0.150b
0	2 (1.4)	0 (0.0)	2 (5.0)	
1	138 (97.2)	100 (98.0)	38 (95.0)	
2	2 (1.4)	2 (2.0)	0 (0.0)	
**Clinical stage, n (%)**				0.007b
IIIA	46 (32.4)	26 (25.5)	20 (50.0)	
IIIB	62 (43.7)	46 (45.1)	16 (40.0)	
IIIC	34 (23.9)	30 (29.4)	4 (10.0)	
**T stage, n (%)**				0.456b
T1	12 (8.5)	10 (9.8)	2 (5.0)	
T2	32 (22.5)	20 (19.6)	12 (30.0)	
T3	26 (18.3)	18 (17.6)	8 (20.0)	
T4	72 (50.7)	54 (52.9)	18 (45.0)	
**N stage, n (%)**				<0.001b
N0	6 (4.2)	2 (2.0)	4 (10.0)	
N1	18 (12.7)	10 (9.8)	8 (20.0)	
N2	68 (47.9)	44 (43.1)	24 (60.0)	
N3	50 (35.2)	46 (45.1)	4 (10.0)	
**Histology, n (%)**				0.720b
Squamous cell carcinoma	78 (54.9)	54 (52.9)	24 (60.0)	
Adenocarcinoma	34 (23.9)	26 (25.5)	8 (20.0)	
Other/NOS	30 (21.1)	22 (21.6)	8 (20.0)	
**Treatment response, n (%)**				–
Partial response (PR)	102 (71.8)	–	–	
Stable disease (SD)	36 (25.4)	–	–	
Progressive disease (PD)	4 (2.8)	–	–	
**PFS, months**				<0.001c
Median (range)	15.5 (3.7–49.2)	19.6 (4.8–49.2)	9.9 (3.7–28.7)	
PFS event, n (%)	98 (69.0)	62 (60.8)	36 (90.0)	–
**CBCT scans per patient**				0.253c
Mean ± SD	3.8 ± 1.0	3.8 ± 1.0	3.9 ± 1.1	
Median (range)	4 (1–7)	4 (2–7)	4 (1–6)	

The *p*-value for treatment response is not applicable because this variable defines the response/non-response grouping; PFS event status is reported descriptively because PFS is a time-to-event outcome and is compared between groups using PFS measured in months. All 142 patients had complete imaging data and were included in the analysis.

^a^ Independent samples *t*-test.

^b^ Chi-squared test or Fisher’s exact test.

^c^ Mann–Whitney *U* test. Best results in bold.

### 2.2 Treatment planning and imaging protocol

All patients underwent CT simulation using a large-bore CT scanner in the treatment position with individualized immobilization. The planning CT images used for analysis were reconstructed with a 5.0-mm slice thickness. Gross tumor volume (GTV) and clinical target volume were delineated by the treating radiation oncologist based on simulation CT, available diagnostic imaging, pathological diagnosis, and clinical staging information. Treatment fields covered the primary tumor and clinically involved nodal regions according to institutional practice. Detailed nodal-region-level irradiation records and target-volume summaries were not systematically available in the retrospective dataset.

The available treatment records provided planned prescription dose information. Patients generally received conventionally fractionated thoracic radiotherapy, with planned total prescription doses ranging from 50 to 66 Gy and most commonly centered approximately 60 Gy. Because actual delivered dose records and treatment interruptions were not uniformly available in the retrospective dataset, planned prescription dose was reported rather than delivered dose. Three-dimensional dose distributions were calculated using the treatment planning system (Eclipse) and exported in DICOM RT Dose format at the same spatial resolution as the planning CT.

CBCT images were acquired using an on-board imaging system integrated with the linear accelerator (Varian TrueBeam or equivalent) for image-guided radiotherapy. CBCT was performed before each treatment fraction for patient positioning verification. Acquisition parameters included 125 kVp, approximately 268 mAs (15 mA tube current with an exposure time of approximately 17.9 s), half-fan acquisition mode, and a reconstruction matrix of 512 × 512, yielding an in-plane pixel spacing of approximately 0.91 mm and a slice thickness of approximately 2.0 mm. This study focused on early CBCT images acquired within 1–2 weeks after treatment initiation, representing the earliest available on-treatment imaging assessment; the first such scan is hereafter referred to as the first on-treatment CBCT (CBCT_1_). In this retrospective dataset, only CBCT scans with complete retrievable DICOM image data and corresponding clinical records were included for analysis, yielding 540 analyzable CBCT scans across all patients, with a median of 4 scans per patient (range: 1–7).

All CBCT images were resampled to match the spatial resolution and coordinate system of the planning CT by first standardizing all images to LPS (left-posterior-superior) orientation, then performing direct resampling to the planning CT coordinate system using SimpleITK with linear interpolation and a default pixel value of −1,000 HU. This approach avoided the need for separate deformable image registration, as CBCT images are inherently acquired in the same coordinate frame as the planning CT within the image-guided radiotherapy workflow. Visual inspection of all registered images was performed to verify spatial alignment. The GTV delineated on the planning CT was propagated to all CBCT images without modification to avoid variability introduced by repeated segmentation. CBCT was used in this study for early imaging phenotype extraction rather than for direct manual measurement of exact tumor shrinkage. Because CBCT has lower soft-tissue contrast than diagnostic CT and may be affected by atelectasis, obstructive inflammation, scatter artifacts, and treatment-related inflammatory changes, the propagated GTV on CBCT should not be interpreted as an exact tumor boundary. Accordingly, CBCT-derived features were interpreted as quantitative measurements within the planned tumor region after image registration.

### 2.3 Feature extraction strategy

From the planning CT, we extracted 107 radiomic features within the GTV using PyRadiomics, spanning three conceptual families: shape descriptors (e.g., voxel and mesh volume, surface area, surface-to-volume ratio, sphericity, principal axis lengths, and maximum 2D/3D diameters), first-order intensity statistics (e.g., mean, standard deviation, percentiles, interquartile range, energy, entropy, uniformity, skewness, and kurtosis of Hounsfield units), and gray-level texture features from the GLCM, GLDM, GLRLM, GLSZM, and NGTDM matrices. This comprehensive pool is subsequently pruned by the within-fold selection pipeline described in Section 2.4 to guard against the curse of dimensionality. From the 3D dose distribution, we computed 10 dose-volume histogram (DVH) parameters within the GTV: the mean, minimum, maximum, and standard deviation of the GTV dose; the dose delivered to 2%, 5%, 50%, 95%, and 98% of the GTV volume (D2, D5, D50, D95, D98); and the dose homogeneity index defined as (D5 − D95)*/*D50.

A critical methodological question in longitudinal imaging analysis is how to represent temporal CBCT information. We systematically evaluated three strategies and adopted a hybrid approach that avoids systematic CT–CBCT bias while preserving treatment-induced dynamics. The first strategy computed delta features between each CBCT and the planning CT, but CBCT images exhibit systematic HU bias from scatter artifacts and beam hardening, introducing artifactual differences unrelated to biological change. The second strategy computed sequential deltas between consecutive CBCTs, but the first CBCT delta still contained systematic CT–CBCT bias. The adopted hybrid approach is formalized below.

#### 2.3.1 First on-treatment CBCT static features

For the first on-treatment CBCT (*i* = 1), absolute measurements are extracted directly within the GTV mask Ω, treating it as a new imaging baseline independent of the planning CT and thereby circumventing systematic HU differences. Let *I*_1_(*v*) denote the voxel intensity and *D*(*v*) the local dose at voxel *v*. The static feature vector $f_{1}^{\text{s}}\in {\mathbb{R}}^{15}$ includes:

- *Volume*: *V*_1_ = |Ω| · Δ*v*, where |Ω| is the voxel count and Δ*v* the voxel volume.
- *First-order intensity statistics*: mean $\mu_{1}=\frac{1}{\left|\Omega \right|}\sum_{v\in \text{Ω}}I_{1}\left(v\right)$, standard deviation $\sigma_{1}={\left[\frac{1}{\left|\Omega \right|}\sum_{v\in \text{Ω}}{\left(I_{1}\left(v\right)-\mu_{1}\right)}^{2}\right]}^{1/2}$, median, minimum, maximum, and interquartile range of HU values, with the interquartile range computed from the 25th and 75th percentiles.
- *Texture descriptors*: entropy $H_{1}=-\sum_{b}p_{1}\left(b\right)\log_{2}p_{1}\left(b\right)$, where *p*_1_(*b*) is the normalized histogram bin frequency; energy $E_{1}=\sum_{v\in \text{Ω}}I_{1}{\left(v\right)}^{2}$ computed over raw HU values; and uniformity $U_{1}=\sum_{b}{\left[p_{1}\left(b\right)\right]}^{2}$ computed over the normalized histogram.
- *Higher-order statistics*: skewness $\gamma_{1}=\frac{1}{\left|\Omega \right|}\sum_{v\in \text{Ω}}{\left[\left(I_{1}\left(v\right)-\mu_{1}\right)/\sigma_{1}\right]}^{3}$ and excess kurtosis $\kappa_{1}=\frac{1}{\left|\Omega \right|}\sum_{v\in \text{Ω}}{\left[\left(I_{1}\left(v\right)-\mu_{1}\right)/\sigma_{1}\right]}^{4}-3$.
- *Dose-weighted features*: dose-weighted mean HU $\mu_{1}^{\text{DW}}=\sum_{v\in \text{Ω}}D\left(v\right)\;I_{1}\left(v\right)/\sum_{v\in \text{Ω}}D\left(v\right)$, dose-weighted standard deviation, and mean dose–HU product, which capture the interaction between dose deposition and tissue density.

#### 2.3.2 Sequential delta features

For subsequent CBCTs (*i* ≥ 2), interscan changes are computed to capture treatment-induced tumor dynamics while avoiding CT–CBCT systematic bias:


$$\text{Δ}f_{j}^{\left(i\right)}=f_{j}^{\left(i\right)}-f_{j}^{\left(i-1\right)},\;i=2,3,\ldots ,n_{\text{cbct}}$$

where $f_{j}^{\left(i\right)}$ is the *j*-th feature from the *i*-th CBCT scan. When *n*_cbct_ ≥ 3 time points are available, temporal summary statistics are derived from the delta sequence $\left\{\text{Δ}f_{j}^{\left(i\right)}\right\}$ as shown in Equations 1–3:

(1) $$\text{Velocity}:\;v_{j}=\left(f_{j}^{\left(n_{\text{cbct}}\right)}-f_{j}^{\left(1\right)}\right)/\left(\tau_{n_{\text{cbct}}}-\tau_{1}\right),$$

(2) $$\text{Slope}:\;\beta_{j}=\frac{\sum_{i=2}^{n_{cbct}}(\tau_{i}-\bar{\tau })\left(\Delta f_{j}^{\left(i\right)}-\bar{\Delta f_{j}}\right)}{\sum_{i=2}^{n_{cbct}}{\left(\tau_{i}-\bar{\tau }\right)}^{2}},$$

(3) $$\text{Volatility}:\;\sigma_{j}^{\text{Δ}}={\left[\frac{1}{n_{cbct}-2}\sum_{i=2}^{n_{\text{cbct}}}{\left(\text{Δ}f_{j}^{\left(i\right)}-\bar{\text{Δ}f_{j}}\right)}^{2}\right]}^{1/2},$$

where *τ_i_* denotes the acquisition time of the *i*-th scan, and $\bar{\tau }$ and $\bar{\text{Δ}f_{j}}$ are the means of $\tau_{i}$ and $\text{Δ}f_{j}^{\left(i\right)}$ over $i=2,\ldots ,n_{\text{cbct}}$, respectively. The cumulative feature set for the primary analysis (*n*_cbct_ = 1) thus comprised 20 planning CT features, 10 dose features, and 15 CBCT_1_ static features, totalling *d* = 45 features. For incremental configurations (*n*_cbct_ ≥ 2), the input dimensionality grows accordingly as interscan delta features and, when at least three time points are available, the temporal summary statistics defined above are appended.

### 2.4 Multitask learning framework

**Figure 1:**
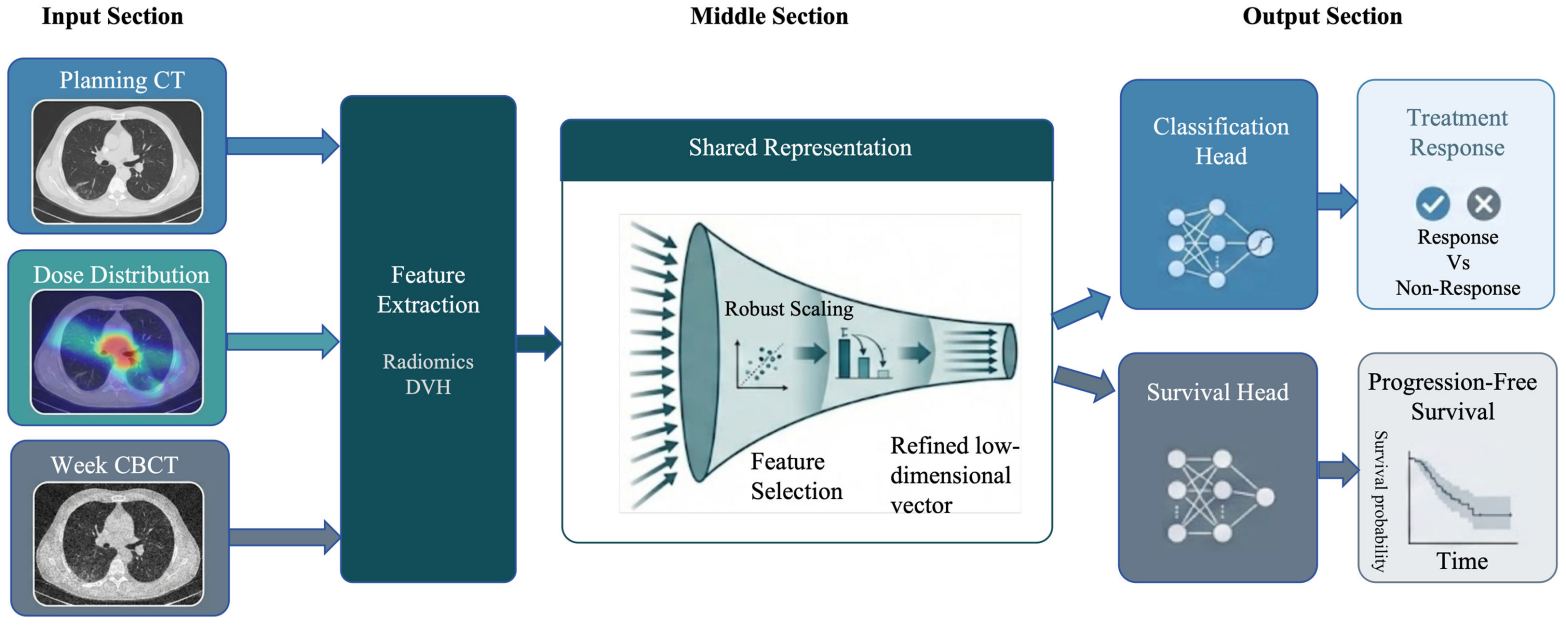
Overview of the proposed multitask learning framework. Multisource features from planning CT, dose distribution, and CBCT are independently embedded, fused via cross-modal attention, and passed through a shared encoder before branching into classification and survival prediction heads.

We propose a lightweight deep learning network that jointly predicts treatment response and progression-free survival in an end-to-end manner. As illustrated in **Figure 1**, the architecture consists of four stages: (1) source-specific embedding, where each input modality (planning CT radiomics, dosimetric features, and CBCT features) is independently projected into a common latent space via dedicated linear layers with layer normalization; (2) cross-modal attention fusion, where a gated attention mechanism learns patient-specific modality importance weights and produces a unified multimodal representation; (3) a shared encoder that refines the fused representation into task-agnostic latent features; and (4) two task-specific prediction heads —a classification head outputting treatment response probability and a survival head outputting a Cox log-risk score. The entire network is trained with a composite loss that couples binary cross-entropy and Cox partial log-likelihood, enabling gradient flow across both tasks through the shared layers.

Formally, let $D={\left\{\left(x_{i},y_{i},t_{i},\delta_{i}\right)\right\}}_{i=1}^{N}$ denote the dataset of *N* = 142 patients, where $x_{i}\in {\mathbb{R}}^{d}$ is the *d*-dimensional multisource feature vector (*d* = 45), $y_{i}\in \left\{0,1\right\}$ is the binary treatment response label, *t_i_ >* 0 is the observed time (PFS or censoring time), and $\delta_{i}\in \left\{0,1\right\}$ is the event indicator (*δ_i_* = 1 if progression observed). The multisource feature vector is constructed as $x_{i}=\left[x_{i}^{\text{CT}},x_{i}^{\text{Dose}},x_{i}^{\text{CBCT}}\right]$, where $x_{i}^{\text{CT}}$, $x_{i}^{\text{Dose}}$, and $x_{i}^{\text{CBCT}}$ represent planning CT radiomic, dosimetric, and first on-treatment CBCT features, respectively. The detailed formulation of each component is described below.

#### 2.4.1 Preprocessing

Prior to network input, each feature dimension is normalized by a robust standardization operator $\phi :{\mathbb{R}}^{d}\to {\mathbb{R}}^{d}$, defined as $\phi {\left(x\right)}_{j}=\left(x_{j}-{\text{median}}_{j}\right)/{\text{IQR}}_{j}$, where median*_j_* and IQR*_j_* are computed exclusively on training data within each cross-validation fold. This interquartile range scaling provides robustness to outliers that are common in radiomic feature distributions. Missing values are imputed with training-set medians prior to standardization.

#### 2.4.2 Feature selection

**Figure 2:**
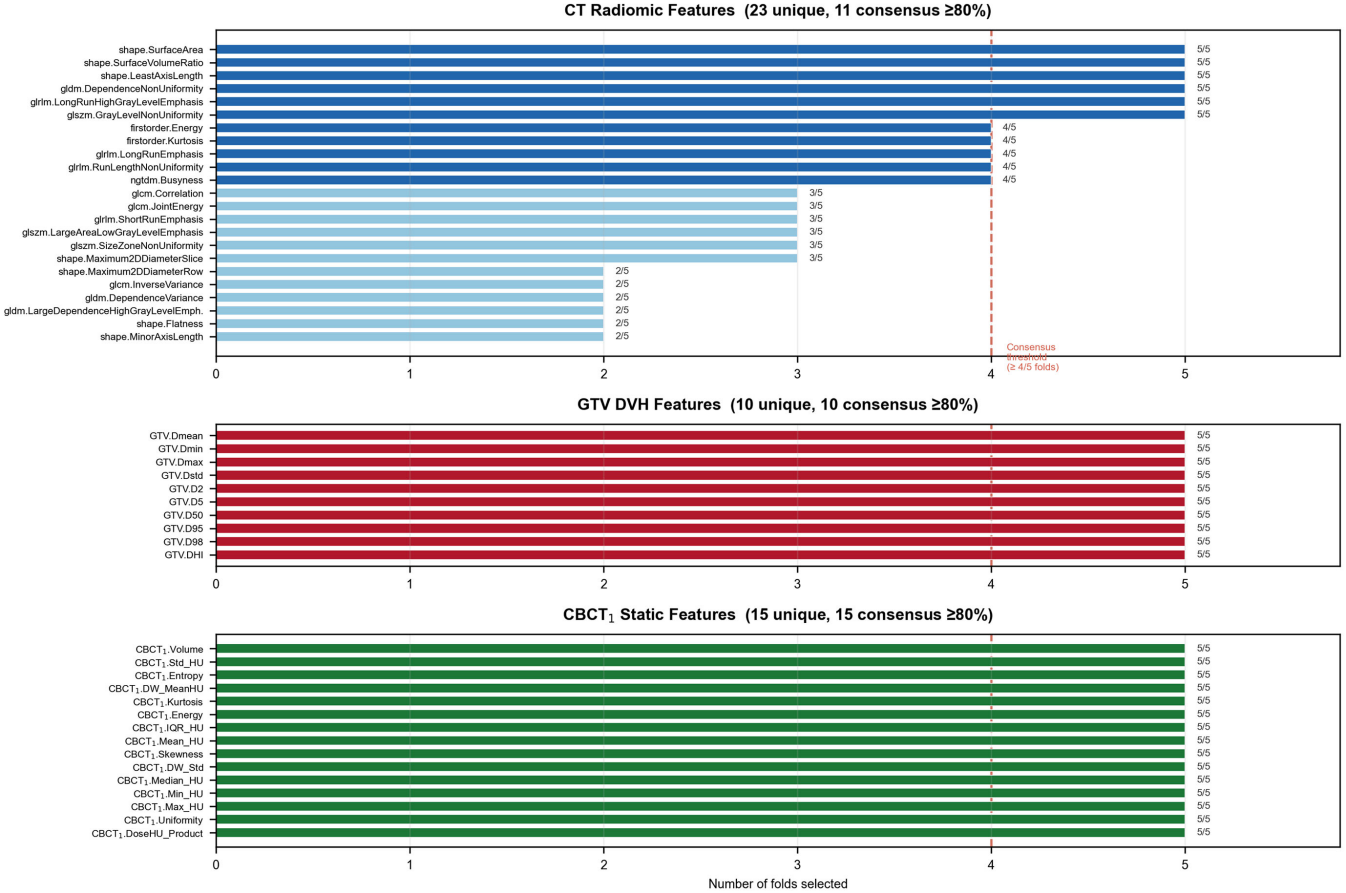
Cross-fold feature selection stability for the primary 45-dimensional input. Horizontal bars indicate the number of folds (out of 5) in which each feature was retained by the within-fold feature-selection pipeline. The dashed red line marks the 80% consensus threshold (≥4/5 folds). CT radiomic features show moderate variability after within-fold pruning and ANOVA ranking, whereas the 10 GTV DVH features and 15 CBCT_1_ static features were consistently retained as compact modality-specific inputs.

For the primary first on-treatment CBCT analysis, the candidate feature set comprised 107 planning CT radiomic features, 10 GTV-based DVH parameters, and 15 CBCT_1_ static features. To mitigate the curse of dimensionality given the moderate sample size (*N* = 142), a three-step within-fold selection pipeline was applied: (1) near-zero-variance features (std< 0.01 after scaling) were removed; (2) among highly correlated feature pairs (Pearson |r|>0.90), the feature with the lower univariate association with the treatment response label was discarded; (3) the remaining candidates were ranked per modality by ANOVA F-statistic (SelectKBest), retaining 20 CT, 10 dosimetric, and 15 CBCT_1_ features within each fold. This yielded a compact 45-dimensional input vector $x_{i}\in {\mathbb{R}}^{45}$ while preserving source-specific discriminative information. Feature selection was performed exclusively on training data within each cross-validation fold to prevent information leakage. In particular, the ANOVA F-statistic computation and feature ranking were repeated independently within every training fold rather than applied once to a globally pre-ranked feature list. Feature-selection stability across the five folds, quantified as the number of folds in which each retained feature was selected, is reported in the Results (Section 3.1, **Figure 2**).

#### 2.4.3 Source-specific feature embedding

To account for the heterogeneous statistical distributions of the three input modalities, each source is independently projected into a common embedding space of dimension *p* as shown in Equations 4–6:

(4) $$h_{i}^{\text{CT}}=\text{ReLU}\left(\text{LN}\left(W_{\text{CT}}x_{i}^{\text{CT}}+b_{\text{CT}}\right)\right),\;W_{\text{CT}}\in {\mathbb{R}}^{p\times 20}$$

(5) $$h_{i}^{\text{Dose}}=\text{ReLU}\left(\text{LN}\left(W_{\text{Dose}}x_{i}^{\text{Dose}}+b_{\text{Dose}}\right)\right),\;W_{\text{Dose}}\in {\mathbb{R}}^{p\times 10}$$

(6) $$h_{i}^{\text{CBCT}}=\text{ReLU}\left(\text{LN}\left(W_{\text{CBCT}}x_{i}^{\text{CBCT}}+b_{\text{CBCT}}\right)\right),\;W_{\text{CBCT}}\in {\mathbb{R}}^{p\times 15}$$

where ReLU(·) = max(0,·), LN(·) denotes layer normalization (28), and *p* = 16 is the embedding dimension. Each modality-specific projection learns its own feature abstraction before cross-modal interaction, preventing scale mismatches between radiomics, dosimetric, and CBCT features.

#### 2.4.4 Cross-modal attention fusion

Rather than simple concatenation, we employ a gated attention mechanism that learns the relative importance of each modality adaptively for each patient. Let S={CT,Dose,CBCT} denote the set of sources. The attention weight for source *s* is shown in Equations 7, 8:

(7) $$e_{i}^{s}=w_{a}^{⊤}\text{tanh }(W_{a}h_{i}^{s}+b_{a}),$$

(8) $$\alpha_{i}^{s}=\frac{exp\;(e_{i}^{s})}{\sum_{s^{\prime }\in S}exp\;(e_{i}^{s^{\prime }})},$$

where $W_{a}\in {\mathbb{R}}^{p\times p}$, $b_{a}\in {\mathbb{R}}^{p}$, and $w_{a}\in {\mathbb{R}}^{p}$ are learnable parameters. As shown in Equation 9, the fused multimodal representation is obtained as the attention-weighted sum.

(9) $$z_{i}^{\text{fused}}=\sum_{s\in S}\alpha_{i}^{s}\;h_{i}^{s}\in {\mathbb{R}}^{p}.$$

This mechanism enables the model to automatically allocate greater weight to the most informative modality on a per-patient basis, providing inherent interpretability of each source’s predictive contribution.

#### 2.4.5 Shared encoder

The fused representation is further refined through a shared fully connected layer that produces task-agnostic latent features as shown in Equation 10:

(10) $$z_{i}=\text{Dropout}\left(\text{ReLU}\left(\text{LN}\left(W_{e}z_{i}^{\text{fused}}+b_{e}\right)\right)\right)\in {\mathbb{R}}^{q},$$

where $W_{e}\in {\mathbb{R}}^{q\times p}$, *q* = 16, and the dropout rate is 0.3. This shared bottleneck ensures that both downstream tasks operate on a common latent space, enabling implicit knowledge transfer between the classification and survival objectives through end-to-end gradient propagation.

##### 2.4.5.1 Task 1: treatment response classification

The classification head maps the shared representation to a treatment response probability through a lightweight subnetwork as shown in Equations 11, 12:

(11) $$h_{i}^{\text{cls}}=\text{ReLU}\left(W_{c1}z_{i}+b_{c1}\right),W_{c1}\in {\mathbb{R}}^{8\times q},$$

(12) $${\hat{y}}_{i}=\sigma \left(w_{c2}^{⊤}h_{i}^{\text{cls}}+b_{c2}\right),$$

where *σ*(·) is the sigmoid function. The classification loss is the weighted binary cross-entropy as shown in Equation 13:

(13) $${ℒ}_{\text{cls}}=-\frac{1}{N}\sum_{i=1}^{N}w_{y_{i}}\left[y_{i}\text{log }{\hat{y}}_{i}+\left(1-y_{i}\right)\text{log }(1-{\hat{y}}_{i})\right],$$

where *w_c_* = *N/*(2*N_c_*) is the inverse-frequency class weight compensating for the imbalance between responders (*N*_1_ = 102) and non-responders (*N*_0_ = 40).

##### 2.4.5.2 Task 2: progression-free survival prediction

The survival head estimates a log-risk score for each patient through a parallel subnetwork, as shown in Equations 14, 15.

(14) $$h_{i}^{\text{surv}}=\text{ReLU}\left(W_{s1}z_{i}+b_{s1}\right),\;W_{s1}\in {\mathbb{R}}^{8\times q},$$

(15) $$r_{i}=w_{s2}^{⊤}h_{i}^{\text{surv}}+b_{s2}.$$

Under the Cox proportional hazards assumption, the hazard function is modeled as *h*(*t* | **z***_i_*) = *h*_0_(*t*)exp(*r_i_*), where *h*_0_(*t*) is the unspecified baseline hazard. The survival loss is the negative Cox partial log-likelihood with Breslow approximation for tied event times as shown in Equation 16:

(16) $${ℒ}_{\text{surv}}=-\frac{1}{\left|ℰ\right|}\sum_{i:\delta_{i}=1}\left[r_{i}-\text{log }\sum_{j\in ℛ\left(t_{i}\right)}\text{exp }(r_{j})\right],$$

where $ℛ\left(t_{i}\right)=\left\{j:t_{j}\geq t_{i}\right\}$ is the risk set at time *t_i_* and $ℰ=\left\{i:\delta_{i}=1\right\}$ is the set of uncensored observations. The risk score exp(*r_i_*) is used directly for concordance evaluation; for clinical risk stratification, patients are dichotomized into high-risk and low-risk groups at the median predicted risk.

#### 2.4.6 Joint optimization

The two tasks are coupled through end-to-end optimization of a composite loss as shown in Equation 17:

(17) $$ℒ=\alpha {ℒ}_{\text{cls}}+\left(1-\alpha \right){ℒ}_{\text{surv}}+\lambda {\left\|\theta \right\|}_{2}^{2},$$

where *α* ∈ [0,1] controls the relative task importance, *λ* is the *ℓ*_2_ regularization strength, and *θ* denotes all learnable parameters. Unlike modular approaches that train task-specific models independently on a shared feature subset, this formulation enables gradients from both classification and survival objectives to flow through the shared encoder and attention fusion layers, encouraging the network to learn a representation that is simultaneously discriminative for treatment response and prognostic for survival. We set *α* = 0.5 and *λ* = 10^−4^. The network is optimized using AdamW (29) with a learning rate of 5 × 10^−4^, weight decay 10^−4^, and (*β*_1_*,β*_2_) = (0.9,0.999). Training proceeds for a maximum of 200 epochs with early stopping (patience = 20) monitored on the combined validation loss. The total number of trainable parameters is approximately 1,750, corresponding to roughly 12 parameters per patient across the full cohort (*N* = 142), which is well within a conservative regime for this sample size and is consistent with the design goal of a lightweight, regularization-friendly architecture.

#### 2.4.7 Cross-validation protocol

Model evaluation follows a 5-fold stratified cross-validation protocol. Let ${\left\{\left(T_{m},V_{m}\right)\right\}}_{m=1}^{5}$ denote the training/validation partitions, with stratification on *y* to preserve class proportions across folds (≈114 training and ≈28 test samples per fold). Within each fold *m*, a strict information isolation protocol is enforced: the robust standardization operator *ϕ*^(^*^m^*^)^ is fitted on $T_{m}$ only, and the entire network (embedding layers, attention, shared encoder, and both task heads) is trained exclusively on standardized training features $\phi^{\left(m\right)}\left(T_{m}\right)$. Test-set predictions are generated by applying *ϕ*^(^*^m^*^)^ to $V_{m}$ and performing a forward pass through the trained network. This protocol ensures that no information from the held-out fold leaks into model training at any stage, providing unbiased estimates of generalization performance.

#### 2.4.8 Implementation and reproducibility

All experiments were implemented in Python 3.10. Radiomic features were extracted with PyRadiomics 3.1.0 using IBSI-compliant feature definitions; prior to feature computation, images were resampled to the planning CT geometry, Hounsfield unit intensities were discretized with a fixed bin width of 25 HU, and identical extraction settings were applied to all modalities and time points. Image input/output and resampling were performed with SimpleITK 2.3.1 (NumPy 1.26.4); feature scaling and selection used scikit-learn 1.3.2; survival baselines used lifelines 0.27.8 and scikit-survival 0.22.2; and the proposed network was implemented in PyTorch 2.1.2 (CUDA 12.1). To ensure reproducibility, a fixed global random seed (seed = 42) was used for cross-validation partitioning, weight initialization, and all stochastic operations, and the five stratified folds were generated once and shared identically across the proposed model and all baselines. Model training was performed on a single NVIDIA H20 GPU (96 GB); owing to the lightweight architecture, end-to-end training required less than 1 min per fold. Source code and feature-extraction definitions are available from the corresponding author upon reasonable request.

## 3 Experimental results

### 3.1 Cohort characteristics

The clinical and demographic characteristics of the 142-patient cohort are summarized in **Table 1**. Based on RECIST 1.1 criteria, 102 patients (71.8%) were classified as responders and 40 (28.2%) as non-responders. The two groups were comparable in age (*p* = 0.166), sex distribution (*p* = 1.000), ECOG performance status, T stage (*p* = 0.456), histological type (*p* = 0.720), and number of CBCT scans (*p* = 0.253). Clinical stage (*p* = 0.007) and N stage (*p*< 0.001) differed significantly between groups, with responders showing a higher proportion of advanced N3 disease and stage IIIC. This imbalance should be interpreted cautiously because response was defined by RECIST-based anatomic change rather than baseline disease severity, and residual confounding may remain in this retrospective cohort. Responders also demonstrated significantly longer PFS than non-responders (median 19.6 vs. 9.9 months, *p*< 0.001).

We also assessed the stability of the within-fold feature-selection pipeline across the five cross-validation folds (**Figure 2**). For CT radiomics, exactly 20 features were retained in each fold; across the five folds, the union of selected CT features contained 23 unique features, of which 11 reached the consensus threshold of being selected in at least four of five folds.

The 10 GTV DVH features and 15 CBCT_1_ static features were retained in all folds because these were predefined compact modality-specific inputs in the primary model.

### 3.2 Experimental setup

To comprehensively evaluate the proposed multitask framework, we conducted comparisons across both treatment response classification and PFS prediction tasks.

For the treatment response classification task, the proposed model was benchmarked against state-of-the-art deep learning methods for multimodal medical imaging: DeepMMIF (30), MDLM (31), M2Fusion (32), DeepRadiomics (33), and DeepSurv (classification head) (20). For the PFS prediction task, the model was evaluated against both classical and deep survival analysis methods: Cox proportional hazards (Cox-PH) (34), L1-penalized Cox-Lasso (35), random survival forest (RSF) (36), gradient boosting survival (GB Survival) (37), MultiDeepsurv (38), Deep-SEA (39), and RobSurv (40).

All methods were evaluated using the same strict 5-fold stratified cross-validation protocol, identical fold partitions, and the same within-fold preprocessing and feature-selection pipeline described in Section 2.4, so that all methods received matched inputs. Hyperparameters for the baseline models were tuned by grid search nested within the training folds. The search grids were as follows: for Cox-Lasso, the *ℓ*_1_ penalty strength over {10^−3^, 10^−2^, 10^−1^, 1}; for random survival forest, the number of trees over {100, 300, 500} and minimum samples per leaf over {3, 5, 10}; for gradient boosting survival, the learning rate over {0.01,0.05,0.1}, number of estimators over {100, 200}, and maximum depth over {2, 3}; and for the deep learning baselines, the learning rate over {10^−4^, 5×10^−4^, 10^−3^}, dropout over {0.2, 0.3, 0.5}, and hidden width over {16, 32}, all optimized with AdamW and early stopping under the same epoch budget as the proposed model. Classification performance was assessed using the area under the receiver operating characteristic curve (AUC), accuracy, sensitivity, and specificity. Survival prediction was evaluated using the concordance index (C-index), hazard ratio (HR) with 95% confidence intervals (CI), and log-rank tests from Kaplan–Meier curves.

### 3.3 Method comparison

#### 3.3.1 Treatment response classification

**Figure 3:**
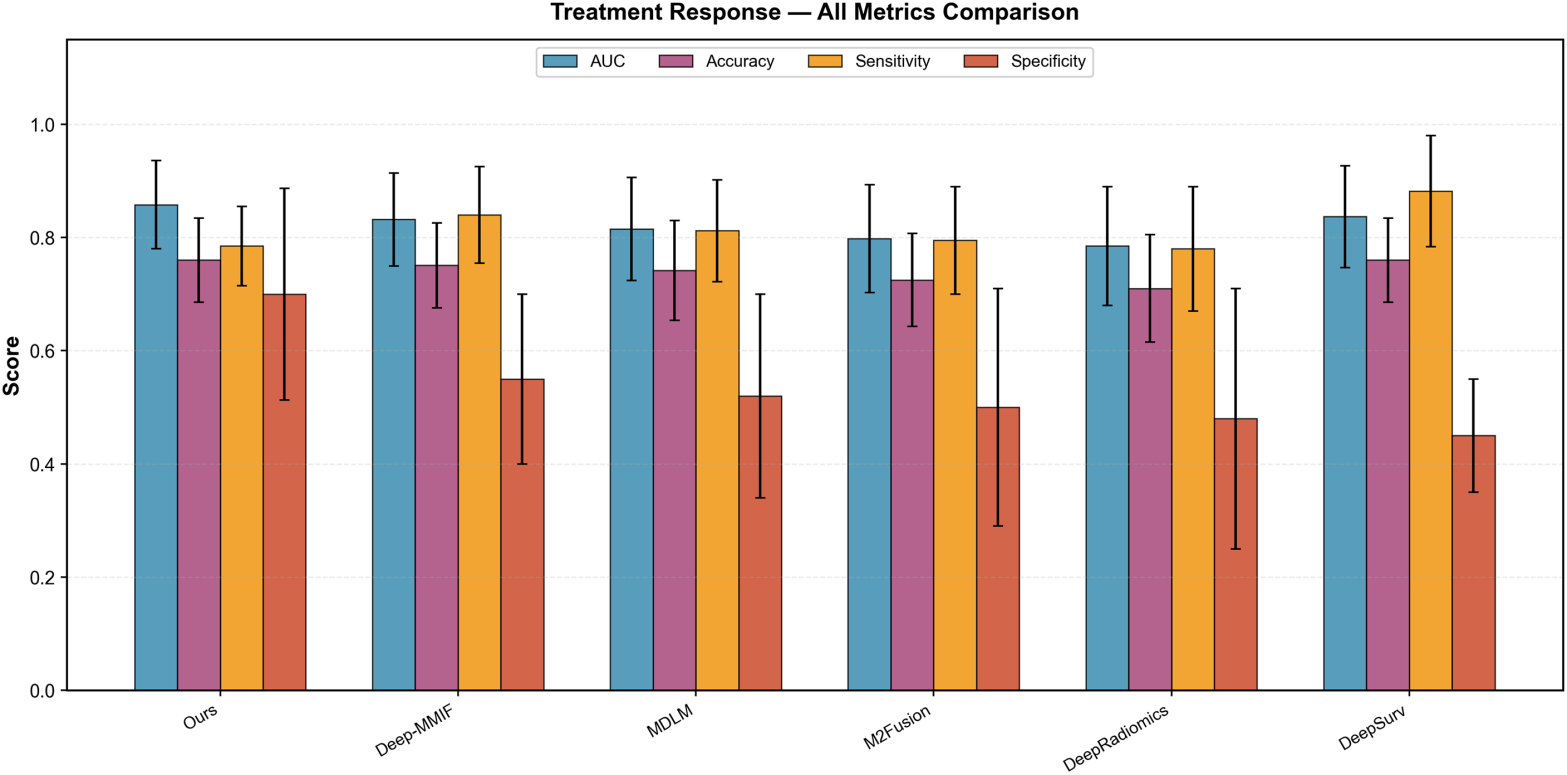
Comparison of classification metrics across the proposed model and state-of-the-art multimodal baselines.

As shown in **Table 2** and **Figure 3**, the proposed model achieved the highest classification performance across all metrics, with an AUC of 0.858 ± 0.078. It outperformed recent multimodal deep learning architectures including Deep-MMIF (AUC 0.832) and MDLM (AUC 0.815), demonstrating that the cross-modal attention fusion mechanism effectively extracts complementary information from planning CT, dose, and first on-treatment CBCT. Notably, while DeepSurv (20) achieved a competitive AUC (0.837) driven by high sensitivity (0.882), its severely imbalanced specificity (0.450) limits its clinical reliability. In contrast, our method maintained well-balanced sensitivity (0.785) and specificity (0.700), yielding the highest overall accuracy.

**Table 2:** Treatment response classification comparison.

Method	AUC	Accuracy	Sensitivity	Specificity
**Ours**	**0.858 ± 0.078**	**0.760 ± 0.074**	0.785 ± 0.070	**0.700 ± 0.187**
Deep-MMIF (30)	0.832 ± 0.082	0.751 ± 0.075	0.840 ± 0.085	0.550 ± 0.150
MDLM (31)	0.815 ± 0.091	0.742 ± 0.088	0.812 ± 0.090	0.520 ± 0.180
M2Fusion (32)	0.798 ± 0.095	0.725 ± 0.082	0.795 ± 0.095	0.500 ± 0.210
DeepRadiomics (33)	0.785 ± 0.105	0.710 ± 0.095	0.780 ± 0.110	0.480 ± 0.230
DeepSurv (20)	0.837 ± 0.090	0.760 ± 0.074	**0.882 ± 0.098**	0.450 ± 0.100

Best results in bold.

#### 3.3.2 Progression-free survival prediction

For PFS prediction, the results are summarized in **Table 3**. The proposed model yielded a superior C-index of 0.672 ± 0.058, outperforming both traditional survival methods (RSF C-index 0.625; Cox-Lasso C-index 0.641) and recent deep learning survival architectures such as MultiDeepsurv (C-index 0.601) and RobSurv (C-index 0.611). Deep learning baselines exhibited notably larger variance (e.g., Deep-SEA std ±0.102), a hallmark of complex models overfitting on limited sample sizes. In contrast, the joint optimization in our multitask framework acts as an implicit regularizer, preserving generalization capability even with *N* = 142.

**Table 3:** Progression-free survival prediction comparison.

Method	C-index	HR (95% CI)	Log-rank p
**Ours**	**0.672 ± 0.058**	**2.45 (1.52–3.95)**	**0.0012**
Cox-PH (34)	0.618 ± 0.072	1.89 (1.18–3.02)	0.0085
Cox-Lasso (35)	0.641 ± 0.065	2.12 (1.33–3.38)	0.0035
RSF (36)	0.625 ± 0.081	1.78 (1.12–2.83)	0.0156
GB Survival (37)	0.632 ± 0.070	1.95 (1.22–3.12)	0.0062
MultiDeepsurv (38)	0.601 ± 0.095	1.65 (1.03–2.64)	0.0382
Deep-SEA (39)	0.587 ± 0.102	1.52 (0.95–2.43)	0.0812
RobSurv (40)	0.611 ± 0.088	1.72 (1.08–2.74)	0.0245

Best results in bold.

**Figure 4:**
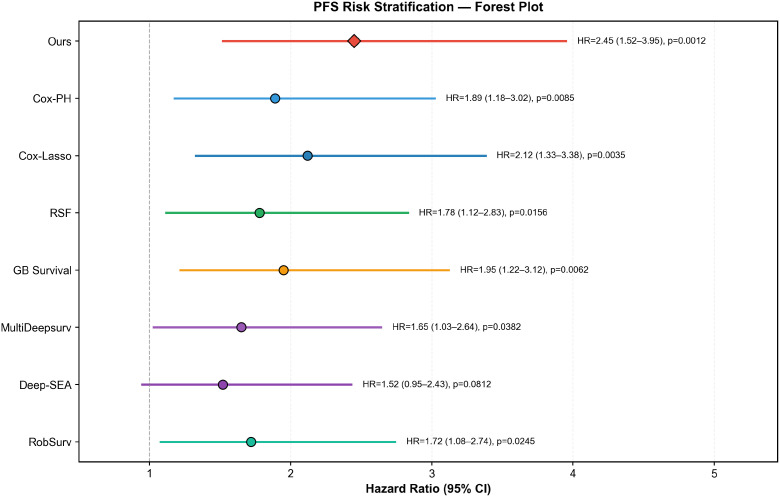
Forest plot of hazard ratios (95% CI) for progression-free survival across evaluated methods.

**Figure 5:**
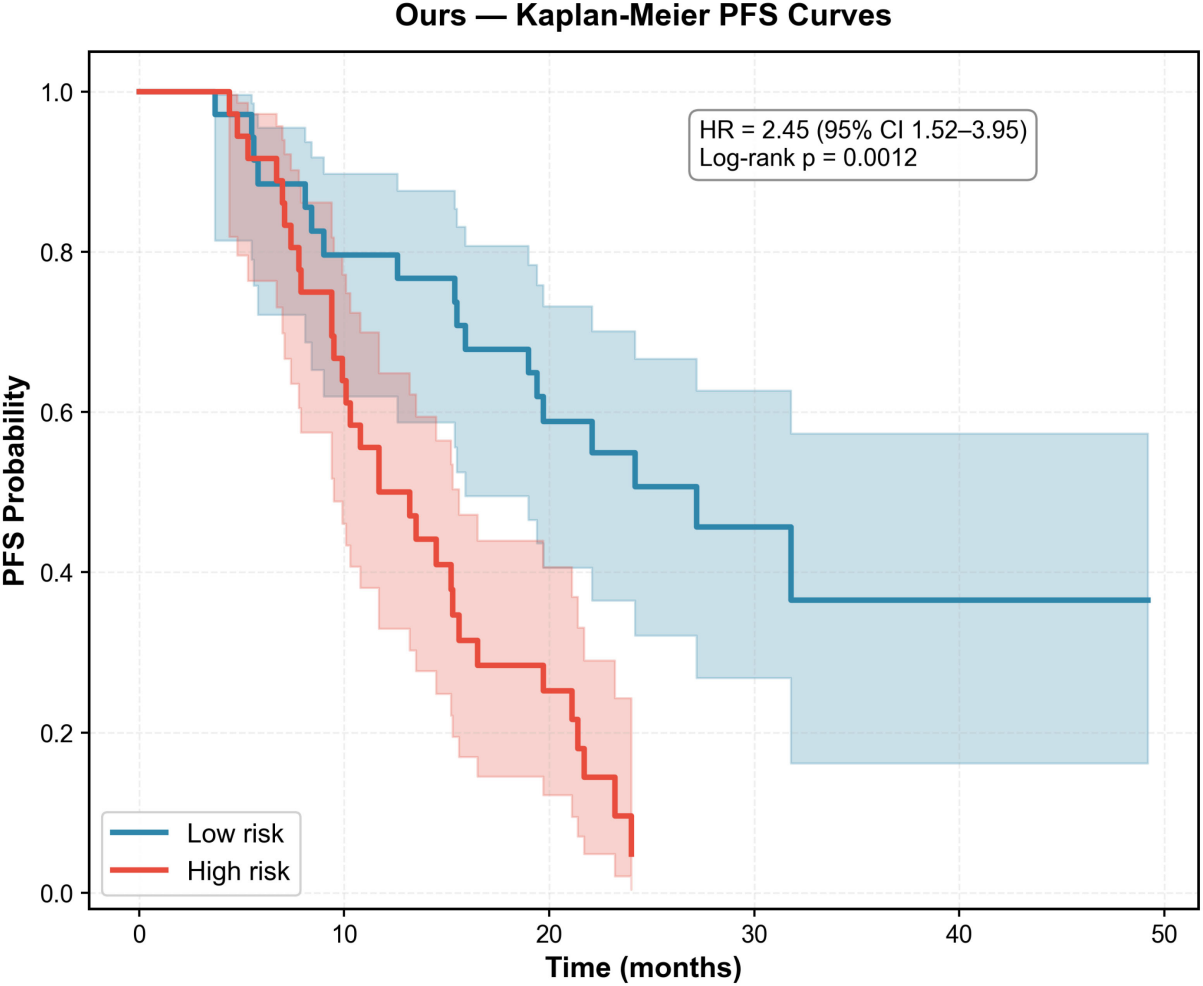
Kaplan–Meier curves for progression-free survival stratified by the proposed model’s predicted risk score (median split).

**Figure 4** presents the hazard ratios for the high-risk versus low-risk groups across all survival methods. The proposed model achieved the largest HR (2.45, 95% CI 1.52–3.95), confirming its superior risk stratification capability. **Figure 5** shows the Kaplan–Meier curves for patients stratified by the median predicted risk score. The median PFS was significantly shorter in the high-risk group (log-rank *p* = 0.0012), supporting the clinical utility of the model for early treatment adaptation decisions.

### 3.4 Ablation study

To quantify the contribution of each modality and architectural component, we performed a two-level ablation analysis on the same 5-fold protocol (**Table 4**). The first level evaluates progressive modality fusion (CT only, CT + Dose, CT + Dose + CBCT_1_). The second level evaluates the impact of task coupling and attention fusion (single-task classifier, multitask without attention, and the full proposed model).

**Table 4:** Ablation study for modality contribution and architecture design.

Configuration	AUC	Accuracy	Sensitivity	Specificity	C-index
CT only	0.788 ± 0.095	0.704 ± 0.083	0.752 ± 0.091	0.600 ± 0.195	0.583 ± 0.089
CT + Dose	0.812 ± 0.088	0.725 ± 0.078	0.774 ± 0.085	0.630 ± 0.172	0.612 ± 0.075
All (ST)	0.838 ± 0.084	0.741 ± 0.075	0.792 ± 0.082	0.650 ± 0.158	0.635 ± 0.071
All (MT, no attn.)	0.847 ± 0.081	0.750 ± 0.073	0.778 ± 0.078	0.680 ± 0.163	0.651 ± 0.064
**All (ours)**	**0.858 ± 0.078**	**0.760 ± 0.074**	**0.785 ± 0.070**	**0.700 ± 0.187**	**0.672 ± 0.058**

All denotes CT + Dose + CBCT_1_. ST = two independent single-task models, MT = multitask joint training. Best results in bold.

The modality-level ablation shows that progressively adding modalities improves predictions: AUC increases from CT only to CT + Dose and further improves after incorporating CBCT_1_. At the architecture level, removing the attention fusion module (C-index 0.651 vs. 0.672) degrades both classification and survival performance, indicating that adaptive modality weighting is beneficial for heterogeneous multisource inputs. Compared with the single-task setting, the multitask formulation consistently improves calibration and specificity, supporting that response and PFS supervision provide complementary regularization in this cohort.

### 3.5 Incremental CBCT analysis

**Figure 6:**
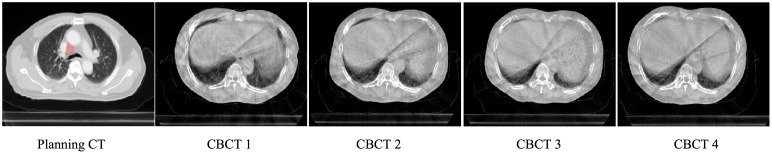
Representative imaging example from a patient with the cohort-median number of CBCT scans. The planning CT panel shows the axial slice with the largest GTV cross-sectional area, with the original planning GTV displayed as a high-visibility contour. Serial CBCT panels show visually corresponding nearby axial slices acquired during radiotherapy. For methodological rigor, the propagated GTV contour is not overlaid on CBCT images because the registered contour should not be interpreted as the exact tumor boundary on CBCT, particularly in the presence of atelectasis, obstructive inflammation, or treatment-related inflammatory changes.

We further investigated whether additional sequential CBCT scans improve prediction by evaluating the model using cumulative features from 0 to 4+ time points (**Table 5**). Adding the first on-treatment CBCT yielded the best classification AUC (0.858) and a strong C-index (0.672). Further CBCT scans progressively degraded classification performance, likely reflecting the increased feature-to-sample ratio and the limited sample size under the current feature-engineering and modeling design. For survival prediction, the C-index showed a marginal improvement up to 3 CBCTs (0.685) before deteriorating, suggesting that longitudinal temporal changes offer minor additional prognostic insights but risk overfitting beyond the optimal temporal window. **Figure 6** shows a representative planning CT and serial CBCT example from a patient with the cohort-median number of CBCT scans.

**Table 5:** Impact of incremental CBCT fractions on classification and survival performance.

CBCT fractions	AUC	C-index
0 (CT + Dose only)	0.812 ± 0.088	0.612 ± 0.075
**+1 CBCT**	**0.858 ± 0.078**	0.672 ± 0.058
+2 CBCT	0.831 ± 0.085	0.679 ± 0.062
+3 CBCT	0.798 ± 0.092	**0.685 ± 0.066**
+4+ CBCT	0.762 ± 0.101	0.668 ± 0.078

Best results in bold.

## 4 Discussion

The proposed framework jointly models treatment response classification and progression-free survival prediction within a unified architecture. When incorporating the first on-treatment CBCT together with planning CT and dose distributions, the model achieved an AUC of 0.858 and a C-index of 0.672.

Our experimental results highlight the value of adaptive feature fusion in multimodal medical imaging. Comparisons with recent state-of-the-art architectures (Deep-MMIF (30), MDLM (31)) suggest that the proposed cross-modal attention mechanism is advantageous in this setting. Because individual patients may exhibit heterogeneous response patterns during radiotherapy, with some showing more informative early anatomical changes on CBCT and others being better characterized by planning CT radiomics or dose distributions, gated attention allows the model to adjust the relative importance of each modality on a per-patient basis. This adaptive weighting may also improve robustness to noisy or less informative inputs, which is supported by the performance reduction observed in the ablation study when attention was removed (C-index decreased from 0.672 to 0.651).

For survival prediction, classical regularized models demonstrated competitive and stable performance. Cox-Lasso (35) achieved the second-highest C-index (0.641) with moderate variance, and GB Survival (37) reached 0.632. These findings indicate that well-regularized linear or ensemble models remain strong baselines in moderate-sample settings. In contrast, specialized deep survival networks such as Deep-SEA (39) and MultiDeepsurv (38) exhibited lower performance and higher variance (e.g., Deep-SEA std 0.102) in this cohort of 142 patients, suggesting that their higher capacity may not translate into consistent gains under limited data conditions. Similarly, although the classification head of DeepSurv (20) achieved a competitive AUC (0.837), it showed markedly imbalanced specificity (0.450), indicating a high false-positive rate. By jointly optimizing a short-term categorical outcome (response) and a long-term temporal outcome (PFS) through a shared encoder, our framework may benefit from implicit cross-task regularization. The learned latent representation is encouraged to capture biologically meaningful patterns relevant to both endpoints, which may help reduce task-specific noise and improve the balance between sensitivity and specificity (0.785/0.700).

A notable finding of this study is that, for treatment response classification, the first on-treatment CBCT yielded better predictive performance than models incorporating additional subsequent CBCT scans, whereas for survival, the concordance index changed only marginally with additional time points. This observation should not be simply interpreted as indicating that later CBCT scans lack clinical value. From a clinical perspective, the first on-treatment CBCT may be particularly informative because it reflects the patient’s actual on-treatment anatomy at the start of radiotherapy, while also capturing initial tumor burden and early treatment-related changes. These characteristics may provide the most direct and robust signal for early prediction. By contrast, subsequent CBCT scans may still contain clinically meaningful complementary information, including the trajectory of tumor regression, treatment-related anatomical adaptation, and normal tissue response during the course of radiotherapy. However, in the present study, the additional discriminative gain from these later time points appeared to be limited for the classification task and did not further improve model performance. A possible explanation is that the accumulation of longitudinal features increased model complexity relative to the available sample size, making the added information more difficult to translate into measurable predictive benefit within the current framework. Therefore, a more appropriate interpretation is that the first on-treatment CBCT captured the major and most robust early predictive signal in this cohort, whereas subsequent CBCT scans provided supplementary rather than dominant information. This conclusion should be confined to the present study setting and should not be generalized to imply that later CBCT imaging has no clinical utility.

From a clinical perspective, the present findings support the feasibility of early risk stratification during radiotherapy using routine on-treatment imaging. Because standard response assessment is typically performed several months after radiotherapy, a model based on early-treatment imaging may provide a meaningful opportunity for earlier identification of patients at higher risk of poor response or progression. Such information could potentially support adaptive treatment strategies or closer monitoring during the treatment course. Nevertheless, the clinical utility of this approach should be further validated before translation into practice.

This study has several limitations. It was a single-institution retrospective study without external validation. CBCT-derived features should be interpreted as early imaging phenotype measurements rather than exact tumor-shrinkage measurements, because tumor boundaries may be affected by atelectasis, inflammation, and limited CBCT soft-tissue contrast. PET/CT, smoking history, molecular biomarkers, detailed delivered-dose information, and nodal/target-volume records were not systematically available. Future studies should validate these findings externally and integrate more complete imaging, treatment, and biomarker data.

## 5 Conclusion

This study introduces a multitask learning framework that jointly predicts treatment response and progression-free survival for lung cancer radiotherapy using multimodal imaging. Comparisons against modern deep learning and classical baseline models suggest that attention-guided joint optimization may help mitigate overfitting in moderate sample sizes. Incremental analysis showed that the first on-treatment CBCT provided the strongest early imaging phenotype signal for response classification in this cohort, whereas adding subsequent scans degraded classification performance and yielded only a marginal change in survival concordance. These findings support the feasibility of early risk stratification using routinely acquired CBCT, while future work should prioritize multi-institutional external validation, systematic PET/CT integration, adaptive tumor assessment, genomic and protein-expression biomarkers, and prospective clinical evaluation.

## Data Availability

The original contributions presented in the study are included in the article/supplementary material. Further inquiries can be directed to the corresponding author.

## References

[1] Bray F Laversanne M Sung H Ferlay J Siegel RL Soerjomataram I . Global cancer statistics 2022: GLOBOCAN estimates of incidence and mortality worldwide for 36 cancers in 185 countries. CA: A Cancer J For Clin. (2024) 74:229–63. doi: 10.3322/caac.21834 38572751

[2] Antonia SJ Villegas A Daniel D Vicente D Murakami S Hui R . Overall survival with durvalumab after chemoradiotherapy in stage III NSCLC. N Engl J Med. (2018) 379:2342–50. doi: 10.1056/nejmoa1809697 30280658

[3] Bradley JD Paulus R Komaki R Masters G Blumenschein G Schild S . Standard-dose versus high-dose conformal radiotherapy with concurrent and consolidation carboplatin plus paclitaxel with or without cetuximab for patients with stage IIIA or IIIB non-small-cell lung cancer (RTOG 0617): a randomised, two-by-two factorial phase 3 study. Lancet Oncol. (2015) 16:187–99. doi: 10.1016/s1470-2045(14)71207-0 25601342 PMC4419359

[4] Goldstraw P Chansky K Crowley J Rami-Porta R Asamura H Eberhardt WEE . The IASLC lung cancer staging project: proposals for revision of the TNM stage groupings in the forthcoming (eighth) edition of the TNM classification for lung cancer. J Thorac Oncol. (2016) 11:39–51. doi: 10.1016/j.jtho.2015.09.009 26762738

[5] Lambin P Leijenaar RTH Deist TM Peerlings J de Jong EEC van Timmeren J . Radiomics: the bridge between medical imaging and personalized medicine. Nat Rev Clin Oncol. (2017) 14:749–62. doi: 10.1038/nrclinonc.2017.141 28975929

[6] Aerts HJWL Rios Velazquez E Leijenaar RTH Parmar C Grossmann P Carvalho S . Decoding tumour phenotype by noninvasive imaging using a quantitative radiomics approach. Nat Commun. (2014) 5:4006. doi: 10.1038/ncomms5006 24892406 PMC4059926

[7] Hosny A Parmar C Coroller TP Grossmann P Zeleznik R Kumar A . Deep learning for lung cancer prognostication: A retrospective multicohort radiomics study. PloS Med. (2018) 15:e1002711. doi: 10.1371/journal.pmed.1002711 30500819 PMC6269088

[8] Avanzo M . Radiomics and deep learning in lung cancer. Strahlenther Onkol. (2020) 196:879–87. doi: 10.1007/s00066-020-01625-9 32367456

[9] Fave X Zhang L Yang J Mackin D Balter P Gomez D . Delta-radiomics features for the prediction of patient outcomes in nonsmall cell lung cancer. Sci Rep. (2017) 7:588. doi: 10.1038/s41598-017-00665-z 28373718 PMC5428827

[10] Han X Wang Y Jia X Zheng Y Ding C Zhang X . Predictive value of delta-radiomic features for prognosis of advanced NSCLC patients undergoing ICI therapy. Sci Rep. (2024) 14:11225. doi: 10.21037/tlcr-24-7 38973966 PMC11225045

[11] Xie D Yu J He C Jiang H Qiu Y Fu L . Predicting the immune therapy response of advanced non-small cell lung cancer using delta radiomics. Front Med. (2025) 12:1541376. doi: 10.3389/fmed.2025.1541376 40248083 PMC12003267

[12] Liu H Schaal D Curry H Clark R Magliari A Kupelian P . Review of cone beam computed tomography based online adaptive radiotherapy: current trend and future direction. Radiat Oncol. (2023) 18:144. doi: 10.1186/s13014-023-02340-2 37660057 PMC10475190

[13] van Timmeren JE van Elmpt W Leijenaar RT Reymen B Lambin P . Survival prediction of non-small cell lung cancer patients using radiomics analyses of cone-beam CT images. Radiother Oncol. (2017) 123:363–9. doi: 10.1016/j.radonc.2017.04.016 28506693

[14] Chang C-W Wang T Qiu RLJ Li X Cammin J Yang K . Patient outcome prognosis for external beam radiation therapy using CBCT-based radiomics: a systematic review. Biomed Phys Eng Express. (2026) 12:012001. doi: 10.1088/2057-1976/ae308b 41435426 PMC13193058

[15] Ma C Wang X Nie K Xiong Z Xu K Yue N . Recent technical advancements and clinical applications of MR-guided radiotherapy in lung cancer treatment. Front Oncol. (2025) 15:1622060. doi: 10.3389/fonc.2025.1622060 40666105 PMC12259636

[16] Wen Q Zhu J Meng X Ma C Bai T Sun X . The value of CBCT-based tumor density and volume variations in prediction of early response to chemoradiation therapy in advanced NSCLC. Sci Rep. (2017) 7:14650. doi: 10.1038/s41598-017-14548-w 29116100 PMC5676710

[17] Cunliffe A Armato SG III Castillo R Pham N Guerrero T Al-Hallaq HA . Lung texture in serial thoracic computed tomography scans: correlation of radiomicsbased features with radiation therapy dose and radiation pneumonitis development. Int J Radiat Oncol Biol Phys. (2015) 91:1048–56. doi: 10.1016/j.ijrobp.2014.11.030 25670540 PMC4383676

[18] Wei M Ye Q Wang X Wang M Hu Y Yang Y . Early tumor shrinkage served as a prognostic factor for patients with stage III non-small cell lung cancer treated with concurrent chemoradiotherapy. Med (Baltimore). (2018) 97:e0632. doi: 10.1097/md.0000000000010632 29742701 PMC5959434

[19] Kang KH Efird JT Podder TK Zhang Y Dowlati A Machtay M . A pilot study examining the prognostic utility of tumor shrinkage on cone-beam computed tomography (CBCT) for stage III locally advanced non-small cell lung cancer patients treated with definitive chemoradiation. Int J Environ Res Public Health. (2021) 18:3241. doi: 10.3390/ijerph18063241 33801033 PMC8004060

[20] Katzman JL Shaham U Cloninger A Bates J Jiang T Kluger Y . DeepSurv: personalized treatment recommender system using a Cox proportional hazards deep neural network. BMC Med Res Methodol. (2018) 18:24. doi: 10.1186/s12874-018-0482-1 29482517 PMC5828433

[21] Wang W Yang G Liu Y Wei L Xu X Zhang C . Multimodal deep learning model for prognostic prediction in cervical cancer receiving definitive radiotherapy: a multi-center study. NPJ Digital Med. (2025) 8:123. doi: 10.1038/s41746-025-01903-9 PMC12322216 40760164

[22] Cho SJ Cho W Choi D Sim G Jeong SY Baik SH . Prediction of treatment response after stereotactic radiosurgery of brain metastasis using deep learning and radiomics on longitudinal MRI data. Sci Rep. (2024) 14:11085. doi: 10.1038/s41598-024-60781-5 38750084 PMC11096355

[23] Kim S . Deep-CR MTLR: a multi-modal approach for cancer survival prediction with competing risks. Proc Mach Learn Res. (2021) 146:101–16. doi: 10.1201/9781315119694-20

[24] Meng M Gu B Bi L Song S Feng D. D Kim J . DeepMTS: deep multi-task learning for survival prediction in patients with advanced nasopharyngeal carcinoma using pretreatment PET/CT. in IEEE Journal of Biomedical and Health Informatics, (2023) 26(9):4497–507. doi: 10.1109/JBHI.2022.3181791 35696469

[25] Lv D Liu R Wang X Liang T Zhang L Zeng W . A comprehensive multi-task deep learning model for kidney cancer: histological subtyping, clinical staging, and anatomical complexity grading. Eur Radio. (2026) 36:12322. doi: 10.1007/s00330-026-12322-z 41606245 PMC13212381

[26] Rhanoui M . Multi-task deep learning for simultaneous classification and segmentation in cancer imaging. Diagnostics. (2025) 15:34. doi: 10.3390/onco5030034 30654563

[27] Ma Q Meng R Li R Dai L Shen F Yuan J . Multitask deep learning model based on multimodal data for predicting recurrence and survival in cancer. BMC Med Inf Decis Making. (2025) 25:305. doi: 10.1186/s12911-025-03050-3 PMC12143085 40474195

[28] Ba JL Kiros JR Hinton GE . Layer normalization. In: Arxiv Preprint Arxiv:1607.06450 (2016).

[29] Loshchilov I Hutter F . (2019). “ Decoupled weight decay regularization”, in: International Conference on Learning Representations.

[30] Lu S Xiao X Yan Z Cheng T Tan X Zhao R . Prognosis forecast of re-irradiation for recurrent nasopharyngeal carcinoma based on deep learning multi-modal information fusion. IEEE J BioMed Health Inf. (2023) 27:4931–42. doi: 10.1109/jbhi.2023.3286656 37384472

[31] Tian R Hou F Zhang H Yu G Yang P Li J . Multimodal fusion model for prognostic prediction and radiotherapy response assessment in head and neck squamous cell carcinoma. NPJ Digital Med. (2025) 8:25. doi: 10.1038/s41746-025-01712-0 PMC12102330 40410262

[32] Zhang S Du S Sun C Li B Shao L Zhang L . (2024). “ M2Fusion: multi-time multimodal fusion for prediction of pathological complete response in breast cancer”, in: Medical Image Computing and Computer Assisted Intervention – MICCAI 2024 ( Springer), 123–32.

[33] Cai Z Li S Xiong Z Lin J Sun Y . Multimodal MRI-based deep-radiomics model predicts response in cervical cancer treated with neoadjuvant chemoradiotherapy. Sci Rep. (2024) 14:19055. doi: 10.1038/s41598-024-70055-9 39154103 PMC11330439

[34] Cox DR . Regression models and life-tables. J R Stat Society: Ser B (Methodological). (1972) 34:187–202. doi: 10.1007/978-1-4612-4380-9_37 27292794

[35] Simon N Friedman J Hastie T Tibshirani R . Regularization paths for Cox’s proportional hazards model via coordinate descent. J Stat Software. (2011) 39:1–13. doi: 10.18637/jss.v039.i05 27065756 PMC4824408

[36] Ishwaran H Kogalur UB Blackstone EH Lauer MS . Random survival forests. Ann Appl Stat. (2008) 2:841–60. doi: 10.1201/9781003493679-19

[37] Hothorn T Bühlmann P Dudoit S Molinaro A Van Der Laan MJ . Survival ensembles. Biostatistics. (2006) 7:355–73. doi: 10.1093/biostatistics/kxj011 16344280

[38] Mao S Liu J . MultiDeepsurv: survival analysis of gastric cancer based on deep learning multimodal fusion models. Biomedical Optics Express. (2024) 16(1):126–41. 39816158 10.1364/BOE.541570 PMC11729289

[39] Ahmad I Riaz S . Deep-SEA: a deep learning based patient specific multi-modality postcancer survival estimation architecture. Appl Intell. (2024) 54:1–20.

[40] Farooq A Singh A Mishra D Chaudhury S . RobSurv: Vector Quantization-Based Multi-Modal Learning for Robust Cancer Survival Prediction. arXiv. (2025). doi: 10.48550/arXiv.2505.02529

